# Retraction: Transcriptional analysis of the F_0_F_1_ ATPase operon of *Corynebacterium glutamicum* ATCC 13032 reveals strong induction by alkaline pH

**DOI:** 10.1099/mic.0.068692-0

**Published:** 2013-06

**Authors:** Mónica Barriuso-Iglesias, Carlos Barreiro, Fabio Flechoso, Juan F. Martín

This article has been retracted at the request of *Microbiology* because identical bands for the 16S rRNA probe controls in the Northern blots were reported to correspond to experiments using different strains and experimental conditions in articles published in this journal and in *Journal of Bacteriology* over a period of 5 years, i.e. the following:

1. Carlos Barreiro, Eva González-Lavado, Miroslav Pátek & Juan-Francisco Martín (2004). Transcriptional analysis of the *groES*-*groEL1*, *groEL2* and *dnaK* genes in *Corynebacterium glutamicum*: characterization of heat shock-induced promoters. *J Bacteriol* **186**, 4813–4817. doi:10.1128/JB.186.14.4813-4817.2004

2. Carlos Barreiro, Eva González-Lavado, Sven Brand, Andreas Tauch & Juan F. Martín (2005). Heat shock proteome analysis of wild-type *Corynebacterium glutamicum* ATCC 13032 and a spontaneous mutant lacking GroEL1, a dispensable chaperone. *J Bacteriol* **187**, 884–889. doi:10.1128/JB.187.3.884-889.2005

3. Mónica Barriuso-Iglesias, Carlos Barreiro, Fabio Flechoso & Juan F. Martín (2006). Transcriptional analysis of the F_0_F_1_ ATPase operon of *Corynebacterium glutamicum* ATCC 13032 reveals strong induction by alkaline pH. *Microbiology* **152**, 11–21; doi:10.1099/mic.0.28383-0

4. Carlos Barreiro, Diana Nakunst, Andrea T. Hüser, Héctor D. de Paz, Jörn Kalinowski & Juan F. Martín (2009). Microarray studies reveal a ‘differential response’ to moderate or severe heat shock of the HrcA- and HspR-dependent systems in *Corynebacterium glutamicum*. *Microbiology* **155**, 359–372; doi: 10.1099/mic.0.019299-0

Drs Barreiro and Martín take sole responsibility for these instances of data duplication and would like to apologize to the readers, reviewers and editors of both the *Journal of Bacteriology* and *Microbiology*.

doi:10.1099/mic.0.28383-0

